# Presence of Li Clusters in Molten LiCl-Li

**DOI:** 10.1038/srep25435

**Published:** 2016-05-05

**Authors:** Augustus Merwin, William C. Phillips, Mark A. Williamson, James L. Willit, Perry N. Motsegood, Dev Chidambaram

**Affiliations:** 1 Materials Science and Engineering, University of Nevada, Reno 1664 N. Virginia St. Reno, MS0388, NV 89557, USA; 2 Nuclear Chemical Engineering Department, Nuclear Engineering Division Argonne National Laboratory, Argonne, IL 60439, USA.

## Abstract

Molten mixtures of lithium chloride and metallic lithium are of significant interest in various metal oxide reduction processes. These solutions have been reported to exhibit seemingly anomalous physical characteristics that lack a comprehensive explanation. In the current work, the physical chemistry of molten solutions of lithium chloride and metallic lithium, with and without lithium oxide, was investigated using *in situ* Raman spectroscopy. The Raman spectra obtained from these solutions were in agreement with the previously reported spectrum of the lithium cluster, Li_8_. This observation is indicative of a nanofluid type colloidal suspension of Li_8_ in a molten salt matrix. It is suggested that the formation and suspension of lithium clusters in lithium chloride is the cause of various phenomena exhibited by these solutions that were previously unexplainable.

Electrolytic reduction in molten LiCl-Li_2_O electrolytes is commonly used in the conversion of TiO_2_^1^, SiO_2_^2^, Ta_2_O_5_^3^, Nb_2_O_5_^4^, and UO_2_ (oxide nuclear fuel) to their base metals^5^,^6^,^7^,^8^. In several of these processes, notably the reduction of actinide oxides, Li^+^ is unavoidably co-reduced with the desired metal oxide, and as a result, elemental Li (Li) is formed and dispersed into the molten salt electrolyte as the process proceeds^9^,^10^. The dissolution of Li in the electrolyte results in a loss of current efficiency in these processes and therefore is of significant interest. Despite the importance of this dispersion phenomenon, molten solutions of Li and LiCl are not well understood. The interface of Li and molten solutions of LiCl, with and without the presence of either Li_2_O or KCl, has been the subject of extensive research^11^,^12^,^13^,^14^,^15^,^16^,^17^,^18^,^19^,^20^. Even so, a knowledge gap still exists in the understanding of the true nature of the molten solutions. Previous research has demonstrated the following phenomena that seemingly are unexplainable:

- Dispersion of Li in LiCl is associated with the formation of a “metal fog” that cannot be explained by physical dissolution^11^,^12^.
- The reported values of the solubility limit of Li in LiCl measured by different methods of analyses vary significantly and are in disagreement^13^,^14^,^15^,^16^,^17^,^18^,^19^.
- The electrical conductivity exhibited by LiCl-Li solutions under metal saturated conditions is unexpectedly low^19^.
- Electrochemical measurements of Li in the presence of LiCl appear as if the thermodynamic activity of Li is significantly lower than unity^13^.
- An intermediate electrochemical potential, between that of Li|Li^+^ and that of the electrode material, is observed when Li disperses from an electrode^20^.

Previous attempts to explain these properties have led to extensive theoretical research on the existence of “hyperlithiated” compounds, such as Li_2_Cl^20^,^21^,^22^,^23^. While mass spectrometry experiments^23^,^24^ have shown the presence of hyperlithiated compounds *in vacuo*, there has been no evidence of their existence in a fused phase. Alternatively, theoretical work has postulated the formation of lithium dimers, Li_2_, in the molten LiCl matrix, as a rationale for explaining the properties of LiCl-Li^25^,^26^. Recently, suspensions of nanoparticles in other molten salts have been investigated for a wide variety of applications due to their unique physical properties^27^. Similarly, experimental work by Nakajima *et al.* suggested that the dispersion of Li in LiCl is the sum of two separate processes, i.e., physical dissolution and colloidal suspension^15^,^16^,^17^,^18^. In these studies, micron-sized particles of metallic Li were observed in quenched LiCl-Li. However, it also was noted that the metallic species would require an emulsifying agent to be suspended in the ionic fluid. While it was proposed that impurities, such as Li_2_O and Li_3_N, act as emulsifying agents, it also was noted that the concentration of dispersed Li in LiCl was not highly dependent on the concentration of either Li_2_O or Li_3_N.

Validation of the previously discussed hypotheses is experimentally challenging due to the highly reactive nature of molten solutions that contain metallic Li and LiCl. Furthermore, *ex situ* experimental techniques are not reliable because the phase stability of mixtures of LiCl and Li is temperature dependent^13^,^14^. In the current research, we used Raman spectroscopy for the *in situ* characterization of molten mixtures of LiCl, Li_2_O, and Li at 923 K in an attempt to understand the nature of these solutions.

## Results

**Figure 1:**
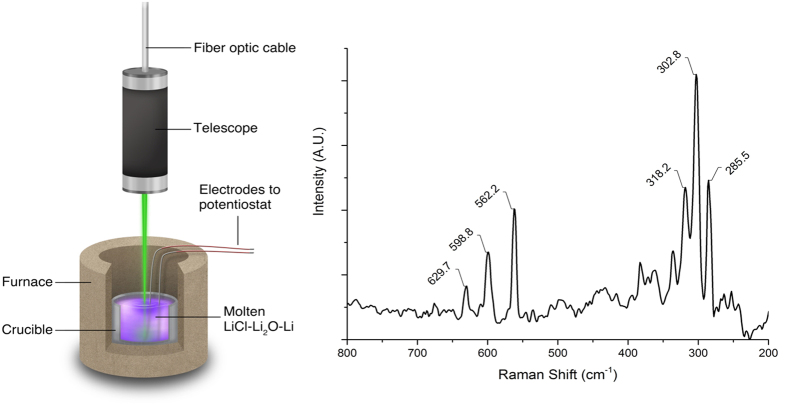
(Left) Schematic diagram of the experimental setup used for the measurement of the Raman features of LiCl-Li_2_O with electrochemically generated Li. (Right) Raman spectrum of LiCl-Li_2_O-Li at 923 K obtained after reducing the equivalent of 1 wt% Li from LiCl-3 wt% Li_2_O. The spectrum was recorded using a 10-mW, 532-nm laser focused vertically onto the surface of the molten solution. The spectrum was comprised of three fundamental features at 285.5, 302.8, and 318.2 cm^−1^, with overtones of decreasing intensity at approximately integer multiples of these Raman shifts.

Figure 1 shows the *in situ* Raman spectrum of molten LiCl-Li_2_O-Li (923 K) after electrochemically reducing an equivalent of 1 wt% of the melt to metallic lithium as well as a schematic depiction of the experimental setup. It was observed that the primary features at 285.5, 302.8, and 318.2 cm^−1^ exhibited overtones of decreasing intensity with increasing Raman shift. Spectra exhibiting the Raman features in Fig. 1 were observed to deviate minimally over the course of 90 minutes. The spectra showed no dependence on the crucible material (Mo and Ta) in which the melts were contained. Additionally, similar Raman spectra were observed in melts of LiCl-Li without the addition of Li_2_O, and the intensity of the spectral features was observed to have a linear dependence on the power of the excitation laser (Supporting Information Figs SI 1–3).

**Figure 2:**
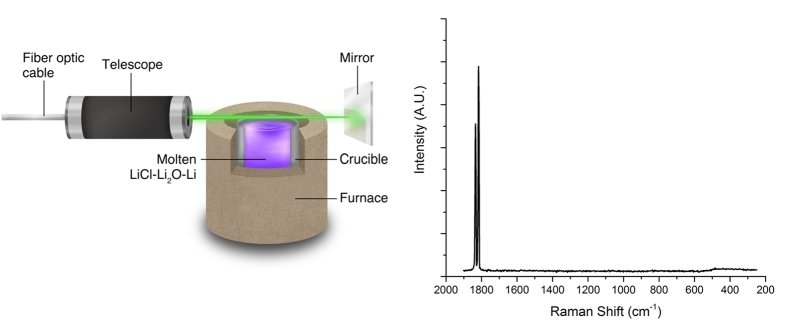
(Left) Schematic depiction of the experimental setup used to characterize the vapor phase that existed above the molten LiCl-Li. The excitation laser was maintained horizontally 5 mm above the surface of the LiCl-Li melt and reflected by a stainless steel mirror. (Right) Recorded spectrum of the vapor existing above the surface of LiCl-Li melt maintained at 923 K. The intense features spanning 1800 to 1900 cm^−1^ are characteristic of the fluorescence of Na from NaCl, which is found as a contaminant in LiCl.

An additional experiment was conducted to confirm that the spectrum shown in Fig. 1 was characteristic of the molten metal/molten salt phase of LiCl-Li_2_O-Li and not of the vapor. To characterize the vapor phase that existed above the mixture, the excitation laser was maintained parallel to the surface of the melt, approximately 5 mm above the fluid/vapor interface, and it was reflected by a stainless steel mirror. The spectrum recorded in this manner, shown in Fig. 2, exhibited high intensity Na fluorescence lines at 1818.1 and 1835.3 cm^−1^ (589 and 589.6 nm)^28^. For the sake of clarity, identical features were omitted from the spectrum in Fig. 1; however, the full spectral range of this spectrum is provided in the Supporting Information (Fig. SI 4).

## Discussions

The Raman spectrum of molten LiCl-Li_2_O-Li at 923 K, shown in Fig. 1, is in agreement with the spectrum of the Li cluster, Li_8,_ previously reported by Kornath *et al.*^29^. The minor discrepancies between the spectra found in the literature and this study are attributed to the difference in the temperatures used in the experiments; the spectra given in this study being recorded 908 K higher than those in the published work. Despite the vast difference in the temperatures used in the experiments, the symmetry of the two spectra were nearly identical and they consisted of three primary features centered on the A_1_ breathing mode of Li_8_ at 302.8 cm^−1^ recorded at 923 K in the current study, and reported at 295.3 cm^−1^ when observed at 15 K. Additionally, the overtones in the spectrum obtained in this study were in agreement with the spectra obtained from Li_8_ by Kornath *et al.*^29^.

Spectroscopy of the vapor phase that exists above the LiCl-Li melt was conducted to confirm that the reported spectrum was a characteristic of the fused phase. The spectrum in Fig. 2, obtained with the laser passing horizontally above the surface of the melt, shows only the fluorescence lines from Na and none of the Raman features that have been suggested to be characteristic of Li_8_. LiCl has a lower free energy of formation than NaCl at 923 K, and, as a result, metallic Li will displace Na from NaCl (found as contaminant in LiCl) in the melt to form LiCl and Na^30^. Furthermore, Na has a vapor pressure of 7.43 kPa at 923 K and is expected to vaporize in significant quantities^31^. Therefore, as a result of the reaction between Li and impurity NaCl, the vapor existing above the LiCl-Li melt consists primarily of Na. Observation of just the Na fluorescence signal in Fig. 2, without the Raman features of interest, demonstrated that the vapor phase does not contain detectable quantities of Li clusters, and that the Raman spectrum shown in Fig. 1 is characteristic of molten LiCl-Li.

Interestingly, the observed Raman spectrum of LiCl-Li does not exhibit the predicted vibrational modes for Li_2_Cl^20^,^21^. However, it was noted that the original work by Hébant *et al.* hypothesized the existence of Li_2_Cl precisely at the interface between liquid Li and LiCl, while the spectrum reported in this study were specifically recorded on the surface of the bulk fluid. Additionally, the Raman mode of the Li_2_ dimer at 349 cm^−1^ was not observed in the molten LiCl-Li^32^,^33^. Li_2_ has been detected under a variety of conditions via Raman spectroscopy, and, previously, it was theorized to be the probable form of Li complexes in molten LiCl-Li^25^,^26^. Importantly, however, the dimer molecule possesses 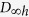 symmetry^34^,^35^, while the Li_8_ cluster possesses a complex hypertetrahedral 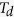 geometry^29^,^36^,^37^,^38^. The axially-symmetric nature of Li_2_ would prevent the dimer from exhibiting a dipole moment and therefore would be immiscible with the pure electrolyte. Alternatively, the asymmetric electronic structure of Li_8_ may enable the suspension of these clusters in an ionic fluid. Colloids of lithium clusters are known to form in solid LiF and Li_2_O crystals when subjected to sufficient irradiation^39^,^40^,^41^. Furthermore, Li_8_ clusters have been observed to be stable in LiF at temperatures up to 1143 K^42^. These reports demonstrated that Li clusters are stable in ionically-bound systems at temperatures exceeding those of the current study.

The variance in reported values of the solubility limit of Li in molten LiCl is suspected to derive from the colloidal suspension of Li clusters in molten LiCl-Li. The solubility limit of Li in LiCl detected by thermal analysis has been reported to be 0.5 ± 0.2 mol% at 913 K^14^, while electrochemical analysis quantified the limit to be 1.8 mol% at 923 K^13^,^19^, and chemical analysis of LiCl-Li quenched at 923 K has been reported to contain greater than 3 mol% Li^15^,^16^,^17^,^18^. Should Li clusters exist in molten solutions of LiCl-Li, a well-defined solubility limit may not exist due to the dispersion mechanism of colloidal suspension in addition to physical dissolution. In this case, the quantity of Li that may be suspended or dispersed under a given set of conditions would be highly dependent upon experimental factors such as thermally induced mixing of the melt or mechanical agitation.

The conglomeration of Li atoms in the form of clusters may explain why the F^−^ center model notably overestimates the electrical conductivity of the LiCl-Li system while accurately predicting such properties for alternative solutions of alkali metal–alkali halide salts^19^,^43^,^44^. The F^−^ center model used to describe electron mobility in metal-salt solutions operates on the assumption that each metal atom acts as an electron donor to the electronic structure of the molten system^45^,^46^,^47^,^48^. Under this assumption, the melt should exhibit a rapid increase in electrical conductivity with the inclusion of a small concentration of electron donor atoms. Alternatively, if Li atoms suspended in molten LiCl-Li form clusters their valance electrons would be confined to the clusters instead of extending into shared electron states of the melt as a whole. This effect can therefore account for the consumption of what would be “free” electrons under metal saturated conditions, resulting in a suppression of the electrical conductivity of the melt^43^,^45^,^46^,^47^,^48^,^49^. Similarly, the F^−^ center model does not apply to polyvalent metal-salt solutions such as Bi-BiI_3_^50^,^51^. In a manner analogous to the proposed formation of Li clusters, metal salt solutions containing transition metals form abnormally reduced complexes referred to as subhalides. The formation of these subhalides localizes what would otherwise be delocalized electrons causing the electron mobility in the melt to be lower than that predicted by the F^−^ center model.

The presence of Li clusters in molten LiCl-Li may additionally elucidate the unattributed electrochemical phenomena exhibited by these solutions. As mentioned previously, electrochemical measurements of Li in contact with molten LiCl exhibits two distinct electrochemical potentials^20^,^21^. Furthermore, the alteration to the Li|Li^+^ open circuit potential that occurs with varying concentrations of Li in the melt is not Nernstian; it behaves as if the activity of the reduced form, Li is not unity and changes based on concentration^13^. These facts suggest that molten solutions of LiCl and Li contain additional Li complexes other than LiCl and Li; a hypothesis that has been debated without confirmation for decades^15^,^20^,^21^,^46^,^48^,^50^,^51^,^52^. It was noted that Li clusters possess significantly different ionization potentials than metallic Li^36^,^37^; therefore they are expected to exhibit thermodynamic activity that is different from the metallic phase. As a result, the anomalous electrochemical properties of LiCl-Li can be attributed to the simultaneous existence of multiple, variable-activity Li phases. The multiple electrochemical potentials exhibited by Li, as well as the appearance of Li not maintaining unit activity are hypothesized to be due to the presence Li clusters and metallically bonded Li in physical contact with the molten solution concurrently.

## Methods

Anhydrous 99.999 wt% purity LiCl, 99.9 wt% purity Li_2_O, and 99.9 wt% purity Li were procured from VWR Scientific. Melts were contained in Mo or Ta crucibles, and were maintained at 923 ± 10 K throughout all of the experiments. The experiments were conducted in an Ar atmosphere glove box containing less than 5 ppm O_2_ and 2 ppm H_2_O.

Molten solutions of LiCl-Li_2_O-Li were generated electrochemically via electrolysis of Li_2_O from molten LiCl-3 wt%-Li_2_O. Electrolysis was conducted using a coil of Pt as the working anode and a coil of stainless steel alloy 316L as the cathode at a cell voltage of 3.2 V, analogous to the cell potential utilized in the electrolytic reduction of UO_2_^9^,^52^. Polarization was conducted in this manner until sufficient charge was passed through the cell to reduce an equivalent of 1 wt% of the melt to metallic Li. The electrodes were maintained in the melt for one hour following electrolysis prior to removal for characterization using Raman spectroscopy. The LiCl-Li melts were prepared in subsequent experiments by directly adding metallic Li to molten LiCl. In all cases, Li_2_O and/or Li were added after drying the LiCl at 823 K to remove residual H_2_O and suppress the formation of LiOH.

Raman spectroscopic measurements were conducted *in situ* using a Thermo-Scientific DXR spectrometer with a custom fiber optic probe procured from InPhotonics. A 10 mW, 532 nm laser beam was passed through the fiber optic cable and telescope before being focused on the surface of the melt. The telescope functioned as the incident and receiving optic for the laser light incident on and reflecting off of the molten solution. In alternative experiments, the laser beam was propagated horizontally, approximately 5 mm above the LiCl-Li mixture, and it was reflected by a metallic surface to characterize the vapor phase that existed on top of the melt. The reported spectra were an average of eight consecutively recorded spectra, each of which was recorded for eight seconds^53^.

## Conclusions

The Raman spectra of molten LiCl-Li_2_O-Li and LiCl-Li were recorded *in situ* at 923 K. A Raman active phase forms in the fused salt when Li is electrochemically reduced from Li_2_O in LiCl, and the recorded spectrum is consistent with previously-reported characteristic spectrum of the lithium cluster Li_8_. These spectra were seen to be characteristic of the bulk fluid rather than the vapor phase that existed above the melt, and it was observed to be stable over a 90-minute period. The presence of a colloidal suspension of lithium clusters in the molten salt may explain the anomalous physical behavior of LiCl-Li solutions.

## Additional Information

**How to cite this article**: Merwin, A. *et al.* Presence of Li Clusters in Molten LiCl-Li. *Sci. Rep.* **6**, 25435; doi: 10.1038/srep25435 (2016).

## Supplementary Material

Supplementary Information
