# Supplementary material for: Presence of Li Clusters in Molten LiCl-Li
Source: Sci Rep. 2016 May 5;6:25435. doi: 10.1038/srep25435 (PMC4857075; doi:10.1038/srep25435)
Supplement: Supplementary Information [file srep25435-s1.pdf]

Presence of Li Clusters in Molten LiCl-Li

Augustus Merwin[1], William C. Phillips[1], Mark A. Williamson[2], James L. Willit[2], Perry N. Motsegood[2], and Dev Chidambaram[1,*]

[1]Materials Science and Engineering, University of Nevada, Reno
1664 N. Virginia St. Reno, MS0388, NV 89557

[2]Nuclear Chemical Engineering Department, Nuclear Engineering Division
Argonne National Laboratory, Argonne, IL 60439

[*]dcc@unr.edu

Supporting Information

Supporting Information

The Raman spectra of LiCl-Li_2O-Li were recorded with varying incident laser power between 0 and 10mW to quantify the dependence of the signal on the power of the laser. Figure SI 1 shows the spectra recorded at various laser powers, along with a plot of the intensity of the Raman feature at 302 cm^{-1} as a function of laser power. A linear response was observed with an R-squared value of 0.979.

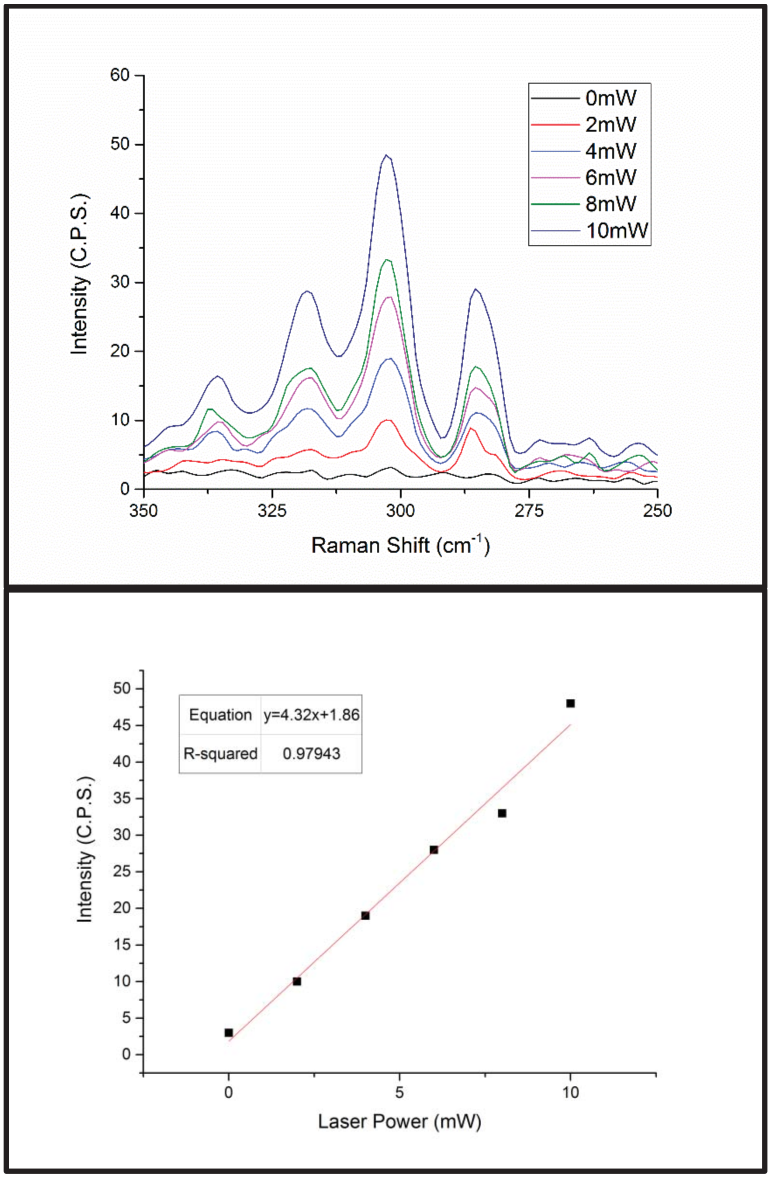

Figure SI 1: (Top) Raman spectra of LiCl-Li at 923 K recorded with an incident laser power increasing from 0 to 10 mW. (Bottom) Plot of the intensity of the 302 cm^{-1} Raman shift as a function of incident laser intensity, showing the linear dependence of signal intensity on the laser power.

To investigate the stability of the LiCl-Li melt as a function of time, Raman spectra of LiCl-Li were recorded 5, 15, 45, and 90 minutes after adding 1-wt% Li to molten LiCl. The spectra recorded at these times are shown in Figure SI 2.

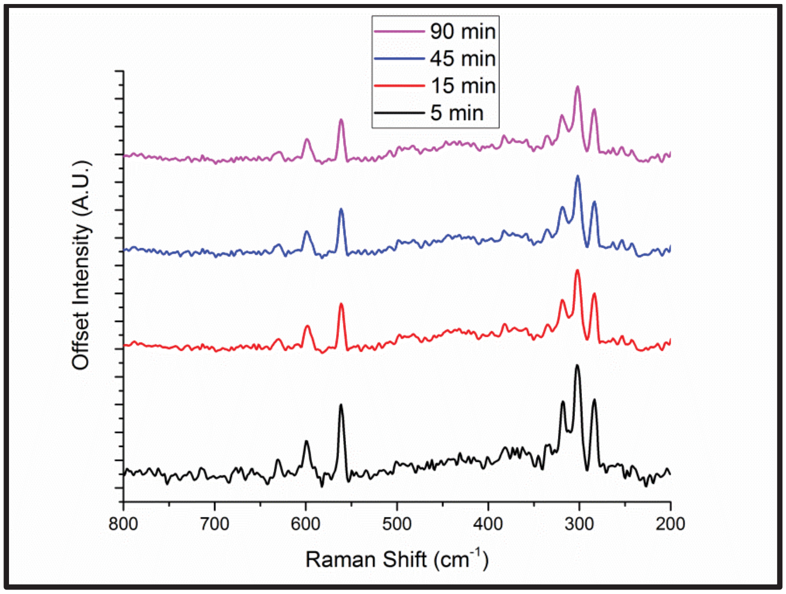

Figure SI 2: Raman spectra of LiCl-Li at 923 K recorded 5, 15, 45, and 90 minutes after adding 1-wt% Li to LiCl. The minimal variation in signal intensity over 90 minutes demonstrates the quasi-stability of the LiCl-Li mixture.

Melts of LiCl-Li were investigated spectroscopically in Mo and Ta crucibles to demonstrate that the observed Raman spectrum of the melt was independent of the crucible material. Raman spectra of LiCl-1-wt% Li recorded in Ta and Mo crucibles are shown in Figure SI 3.

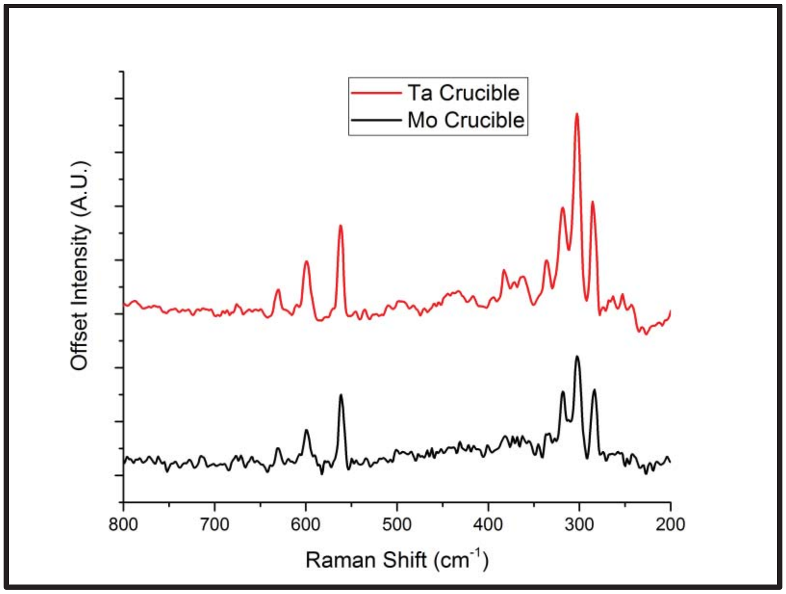

Figure SI 3: Raman spectra of LiCl-Li melt at 923 K contained in Mo and Ta crucibles. The results demonstrate that the observed spectrum is independent of the crucible material.

The Raman spectrum shown in Figure 1 was restricted to a narrow spectral band for clarity. Figure SI 4 shows the spectrum in Figure 1 across a larger spectral range, and it includes both the Raman features attributed to the presence of Li_8 as well as the Na fluoresce lines exhibited in Figure 2.

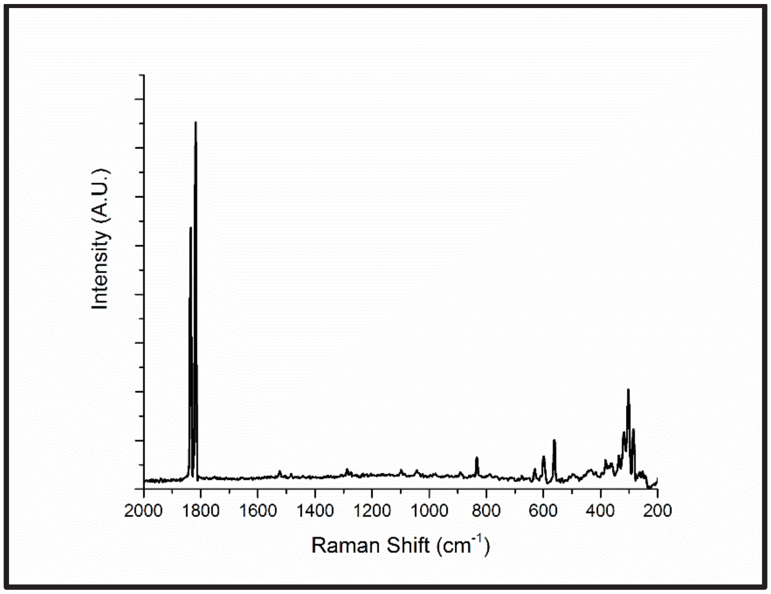

Figure SI 4: Raman spectrum of LiCl-Li_2O-Li at 923 K shown in Figure 1 across a larger spectral range. The spectrum exhibits the Raman modes attributed to the presence of Li_8 as well as Na fluoresce.

The Raman spectrum of molten LiCl-3-wt% Li_2O is shown in Figure SI 5 as a control to demonstrate that solutions that do not contain metallic Li lack detectable Raman activity.

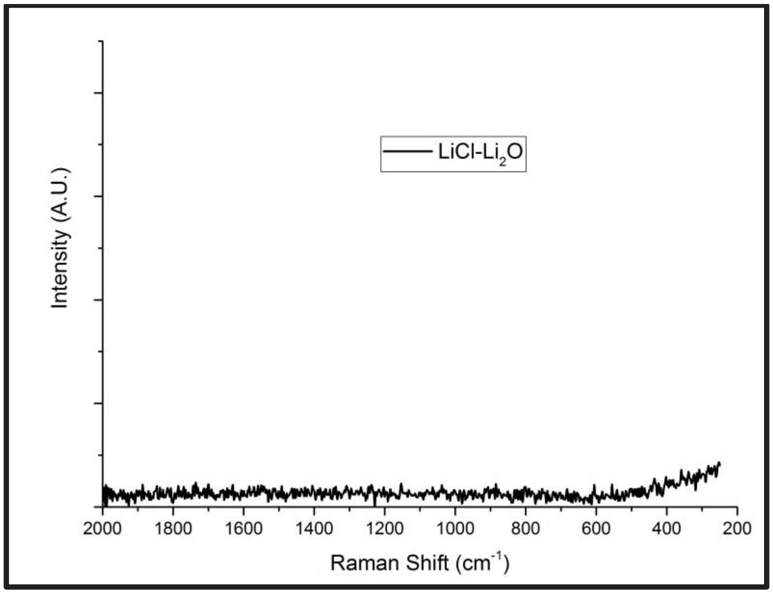

Figure SI 5: Raman spectrum of LiCl-Li_2O at 923 K before the addition of metallic Li to the melt.
